# Autistic-led insights on airport accessibility: A retrospective analysis of environmental assessments

**DOI:** 10.1177/13623613251337200

**Published:** 2025-05-09

**Authors:** Chris Edwards, Abigail MA Love, Ru Ying Cai, Tom Tutton, Emma Beardsley, Vicki Gibbs

**Affiliations:** 1 Aspect (Autism Spectrum Australia), Australia; 2 Griffith University, Australia; 3 La Trobe University, Australia; 4 The University of Sydney, Australia

**Keywords:** accessibility, air travel, airport, autism-friendly, tourism

## Abstract

Autistic people often encounter significant barriers when navigating airports, largely due to overwhelming sensory environments, stringent security procedures, and crowded spaces. This study aimed to enhance understanding of airport accessibility by retrospectively analyzing reports from Autistic-led environmental assessments conducted at six Australian airports between 2017 and 2024. Drawing on an evidence-informed autism-friendly framework, the assessments focused on eight key elements, including sensory adaptations, communication supports, and preparation and predictability. Through reflexive thematic analysis, two key themes were generated: (1) navigating the sensory landscape and (2) help passengers navigate with confidence. Our findings underscore the importance of sensory adjustments—such as natural lighting and quiet spaces—alongside clearer visual aids and communication supports. Importantly, this study represents the first systematic application of autism-friendly principles to airport environments, offering practical, evidence-based recommendations for more inclusive design. By foregrounding the perspectives of Autistic individuals, this research highlights the need for participatory design approaches to create public spaces that are genuinely accessible and supportive for Autistic travelers

**Lay abstract:**

Airports can be challenging for Autistic people because of overwhelming sensory experiences like bright lights and loud noises, security processes, and crowded spaces. This study analyzed reports from six Australian airports, produced between 2017 and 2024, to understand how airports can be made better for Autistic travelers. These reports were based on assessments led by Autistic people and used a specific framework to evaluate areas like sensory experiences and communication needs. The study found two main ways to improve accessibility: (1) reducing sensory challenges, like loud noises or bright, overwhelming areas, and (2) improving communication and wayfinding to make navigation clearer and easier. Some of the recommendations included providing quiet spaces for travelers, using softer and more natural lighting, and improving signs to make it easier for people to navigate airports. This is the first study to apply autism-friendly principles to airports and shows the importance of involving Autistic people in the design of more inclusive public spaces. The findings offer practical recommendations for airports around the world to better support Autistic travelers.

There is growing recognition of the need to create environments that are genuinely inclusive of Autistic^1^ people, who often experience the world through distinct sensory, social, and communicative modalities. Autistic people frequently have heightened or diminished responses to sensory stimuli (e.g. sounds, lights, and textures), which can make environments overwhelming or under-stimulating (Brake, 2024; Chen et al., 2024; MacLennan et al., 2021; Parmar et al., 2021). In addition, social relating and communication styles can differ, with many Autistic people finding it challenging to navigate non-autistic social norms, often leading to misunderstandings or social exclusion (Crompton et al., 2020; Milton, 2012). The increasing awareness of these differences has prompted efforts across various sectors—including healthcare, education, and public spaces—to improve accessibility for individuals who might otherwise face exclusion (e.g. Clouse et al., 2019; Fletcher-Watson & May, 2018; Parmar et al., 2022).

The rise of “autism-friendly” initiatives (e.g. initiatives that aim to make spaces more accessible and inclusive for Autistic people; Manning et al., 2023) underscores an evolving understanding that environments are not neutral but play a critical role in either facilitating or hindering an individual’s ability to engage. How well environments accommodate diverse sensory and cognitive needs can dramatically shape the experiences of Autistic people (MacLennan et al., 2023). As Dwyer (2022) highlights, societal barriers often play a significant role in disabling neurodivergent individuals such as Autistic people, positioning the environment, rather than inherent deficits, as a critical site for intervention. This perspective challenges the traditional medical model, which primarily focuses on addressing individual limitations. In contrast, autism-friendly practices involve reducing environmental barriers, such as minimizing overwhelming sensory input from lighting, noise, or crowds, while promoting enablers like predictable routines, clear communication, and the use of visual aids to help individuals anticipate what to expect (Cunningham, 2022; Edwards, Tutton et al., 2024; Fletcher-Watson & May, 2018; Mostafa, 2021; Parmar et al., 2022; Stogiannos et al., 2023).

Several frameworks have emerged to guide the development of autism-friendly environments, each offering distinct approaches to address sensory and cognitive needs. The Autism ASPECTSS Design Index (Mostafa, 2020) emphasizes sensory zoning, advocating for spaces with controlled acoustics, appropriate lighting, and materials that reduce sensory overload. Its core components offer practical strategies for creating environments that better suit Autistic people with a focus on acoustics, spatial sequencing, escape spaces, compartmentalization, transitions, sensory zoning, and safety. Similarly, the SERVICE principles (Garner et al., 2024) outline a community-centered approach that focuses on adjustments that businesses and public spaces can make at low or no cost. These principles include creating zones with reduced sensory input, ensuring clear communication, and providing respectful, patient customer service. Autistic SPACE (Doherty et al., 2023) takes a more rights-based perspective, emphasizing self-determination and autonomy for Autistic people in healthcare settings by ensuring that spaces are structured to accommodate sensory and social preferences, allowing for flexibility in how spaces are used. Finally, the PAS 6463 Standard (British Standards Institution, 2022) offers broader neurodiversity-related built environment guidance, addressing not only the potential needs of Autistic people but also others with sensory processing differences. This standard recommends incorporating adaptable design elements such as quiet zones, minimizing visual clutter, and ensuring environments are sensory-friendly with predictable layouts to reduce cognitive load and improve accessibility. Together, these frameworks highlight the importance of sensory adaptations, cognitive load reduction, spatial clarity, and fostering empathy and understanding.

MacLennan and colleagues’ (2023) findings complement these frameworks by detailing six principles that describe how public spaces can either support or hinder Autistic people, based on their sensory experiences. Their study identifies the importance of the sensoryscape, which encompasses the type, intensity, and duration of sensory inputs like sounds and lights. In addition, the physical sense of space—whether a place feels open or confined—affects how manageable a sensory environment is. Predictability is also a key factor, as it helps reduce uncertainty and anxiety by ensuring that sensory and environmental cues remain consistent. A critical component is the provision of recovery spaces, where individuals can temporarily retreat to manage sensory overload, further supporting their ability to re-engage with the environment. Importantly, MacLennan et al. (2023) emphasize creating an autism-friendly environment extends beyond sensory adjustments; it also requires an understanding from staff and the public, who play a key role in recognizing and accommodating sensory and social needs to foster a more inclusive and accessible space for Autistic people.

While research on autism-friendly environments in areas like healthcare is growing (e.g. Parmar et al., 2022; Stogiannos et al., 2023), complex public spaces such as airports remain largely unexplored. There is a pressing need to extend autism-friendly research to airports, particularly given the established link between tourism and improved quality of life (Uysal et al., 2016). Airports are uniquely high-stress environments, often marked by overwhelming sensory stimuli, crowded spaces, unpredictability, and the rigid demands of security procedures. For Autistic travelers, these conditions can exacerbate anxiety and distress, creating significant barriers to air travel (Conde et al., 2023; Dempsey et al., 2021; Sedgley et al., 2017; Zhao et al., 2023). Consequently, it is unsurprising that some Autistic people and their families choose to avoid air travel altogether (Dempsey et al., 2021). To address these challenges, various supportive measures have been proposed, including the provision of preparation resources, systems to help bypass crowds or lengthy queues, sensory-friendly or quiet spaces, improved wayfinding, and autism awareness training for airport staff (Cerdan Chiscano, 2021; Conde et al., 2023; Dempsey et al., 2021; Neo & Flaherty, 2019; Santos et al., 2024). However, these are often provided as isolated solutions rather than forming part of a comprehensive, evidence-based approach to improve airport accessibility.

The importance of participatory design in creating accessible environments is crucial, especially when it comes to the needs of Autistic people. As Black et al. (2022) emphasize, Autistic people must be directly involved in design and redesign considerations, as lived experience provides invaluable insights that non-autistic people may overlook. This aligns with the growing recognition that Autistic people are autism experts (Gillespie-Lynch et al., 2017). To date, tourism and travel research has predominantly focused on physical disabilities, often neglecting hidden disabilities like autism (Jepson et al., 2024). This oversight is particularly problematic for complex environments like airports, where solutions developed without Autistic input may fail to address critical barriers or inadvertently create new ones.

Recently, efforts to improve accessibility for Autistic people in public spaces, including airports, have gained momentum. This is particularly critical in Australia, where the vast geography and dependence on air travel for domestic and international connectivity highlight the need for inclusive airport environments (Bureau of Infrastructure and Transport Research Economics, 2021). One notable initiative, the Hidden Disability Sunflower program, seeks to raise staff awareness about people with invisible disabilities (Hidden Disability Sunflower, 2024). By allowing people to subtly signal that they may require support, the program offers a promising step toward greater inclusivity. However, its effectiveness hinges on comprehensive staff training and does not adequately address systemic barriers or the specific challenges faced by Autistic people, such as sensory overload, difficulties with wayfinding, and the need for predictability in stressful environments (Edwards, Love et al., 2024; MacLennan et al., 2023; Tata et al., 2024). While initial research indicates that the Sunflower program can be beneficial when implemented by well-trained staff (Edwards, Love et al., 2024), there remains a critical gap in understanding broader autism-friendly practices within complex public settings like airports.

## Current study

This qualitative study, grounded in a constructionist epistemology, seeks to address the gap in understanding airport accessibility for Autistic travelers by retrospectively analyzing six Autistic-led environmental assessment reports written from 2017 to 2024. Using reflexive thematic analysis (RTA), the study explores recurring themes and patterns to illustrate the shared story of accessibility challenges and enablers across the Australian airports. The findings aim to answer the research question: What are the common accessibility challenges and enablers in airports, as identified through Autistic-led environmental assessments guided by an autism-friendly framework?

## Method

### Ethics approval

Ethical approval for this research was granted by the Griffith University Human Research Ethics Committee (2023/860). The environmental assessment reports analyzed in this study were originally prepared by Autistic consultants (including E.B.) as part of their professional roles with Aspect (Autism Spectrum Australia) between 2017 and 2024. Permission to analyze these reports for research purposes was granted by the Head of Autism Friendly at Aspect (T.T.), who holds the authority to approve the use of organizational materials for research purposes. Importantly, the reports did not contain identifiable personal information, and all data used in this study were anonymized. Confidentiality was strictly maintained throughout the research process, ensuring that neither individual consultants nor airport staff could be identified in the analysis or reporting of findings.

### Airport assessments

Between 2017 and 2024, the Autism Friendly team conducted comprehensive environmental assessments at six domestic and international Australian airports (see Table 1 for a brief description of airports). Each assessment involved a walkthrough conducted by two consultants, at least one of whom was Autistic, drawn from a pool of four Autistic (including E.B.) and one non-autistic consultant (T.T.). While not Autistic himself, the non-autistic consultant has worked closely with Autistic colleagues for over a decade and bases his observations on their shared insights and lived experiences, rather than on personal interpretation. The walkthroughs simulated the experiences of both departing and arriving passengers, allowing consultants to observe sensory, social, and communicative demands at various stages of the airport journey, including pre-travel engagement with the airport website. All six assessments covered the full passenger journey—from pre-travel information through to in-airport experiences—including check-in, security, waiting areas, and boarding.

**Table 1.** Overview of airport environments assessed.

Airport number	Year assessed	Airport description
Airport 1	2023/2024	An airport primarily serving domestic routes with modern infrastructure and accessible design.
Airport 2	2019	An airport serving both international and domestic flights, known for handling a significant number of leisure travelers due to its location near popular tourist destinations.
Airport 3	2024	A large international and domestic airport with a complex layout, serving a high volume of passengers with numerous amenities.
Airport 4	2023	A regional airport providing domestic flights with limited services and facilities, located in a remote area.
Airport 5	2017	A major international and domestic airport with high passenger volumes and a wide range of services and facilities.
Airport 6	2023	A regional airport offering domestic flights, characterized by a quieter environment and straightforward travel experience.

The Aspect Autism Friendly Framework guided the environmental assessments, providing a structured and consistent approach to evaluating airport accessibility for Autistic travelers. Developed by the Autism Friendly team through a co-design process involving Autistic and non-autistic practitioners, the framework is organized around eight key elements that emphasize creating environments that are predictable, structured, and supportive of sensory and communication differences. Observations and experiences are documented contemporaneously through detailed notes and photographs as consultants consider aspects of the environment guided by each of the eight elements, for example, sensory adaptations, communication supports, preparation and predictability. A description of the Aspect Autism Friendly Framework including its development, structure, and application are provided in Supplemental Material.

Following each assessment, the consultants produced an environmental report documenting strengths and areas for improvement—highlighting both positive practices and identified barriers—along with visual documentation (photographs, not included in this study due to ethical considerations) and an evaluation of pre-travel preparation materials, such as airport websites and resources aimed at reducing traveler anxiety. These reports, delivered to each airport as part of a consultancy service, ranged from 15 to 41 pages in length and combined qualitative observations with photographic evidence to illustrate key aspects of the airport environment. While most reports were structured around the framework’s eight key elements, the most recent report (Airport 3) was organized according to the passenger journey. This alternative format—beginning with pre-travel information and progressing through each stage of the airport experience—was adopted to reduce redundancy across framework elements and offer a more intuitive, user-centered structure. Despite this variation in format, all reports addressed the same core accessibility domains, ensuring consistency across assessments for comparative analysis.

### Data analysis

We employed RTA (Braun & Clarke, 2021; Braun et al., 2019) to analyze the environmental assessment reports within NVivo 15. This approach was selected for its flexibility in identifying patterns of meaning and its emphasis on the researcher’s active role in data interpretation. Consistent with the study’s constructionist epistemology, we adopted a primarily inductive approach, creating themes directly from the data rather than being shaped by predefined categories or elements of the Aspect Autism Friendly Framework. This ensured that the analysis was grounded in the consultants’ lived experiences and observations, reflecting their insights and the broader Autistic community’s potential experiences of airport environments.

The analysis process was iterative and recursive, characterized by deep engagement with the data. Familiarization involved close reading and re-reading of the reports, during which initial observations and reflections were noted. Coding was conducted systematically, primarily at the semantic level, to capture explicit features of airport environments influencing accessibility. These codes were revisited, refined, and grouped into themes through an iterative process that involved ongoing reflection and critical discussion. Themes were constructed as patterns of shared meaning organized around central concepts, encapsulating the consultants’ collective narratives on systemic challenges and positive practices across the six airports.

The refinement of themes involved comparing them against the data set to ensure coherence and consistency, as well as reviewing their alignment with the research question and constructionist epistemology. The final themes were reviewed collaboratively to confirm that they meaningfully represented the consultants’ perspectives and extended beyond the parameters of the autism-friendly framework.

### Positionality

This research was conducted by the Aspect Research Centre for Autism Practice (ARCAP), the research arm of Aspect, in collaboration with the Autism Friendly team. The project was led by an Autistic autism researcher with lived experience of air travel, providing valuable insights into accessibility challenges. The research team also included a parent and a sibling of Autistic people, whose perspectives further enriched the understanding of barriers and supports for Autistic travelers. While the Autism Friendly team, which created the environmental assessment reports analyzed in this study, did not directly participate in the data analysis, members of their team informed the research. In particular, the Head of Autism Friendly (T.T.) and the most experienced Autistic consultant (E.B.) provided feedback on the interpretation of findings during the manuscript development phase, contributing to the study’s contextual depth and practical relevance. Guided by Aspect’s core strategy of fostering community understanding about autism to create a more inclusive and accessible world, this work embedded this principle into both the research process and the environmental assessments. This approach ensured the study remained firmly grounded in Autistic perspectives while advancing its commitment to driving meaningful social change.

## Results

**Figure 1.**
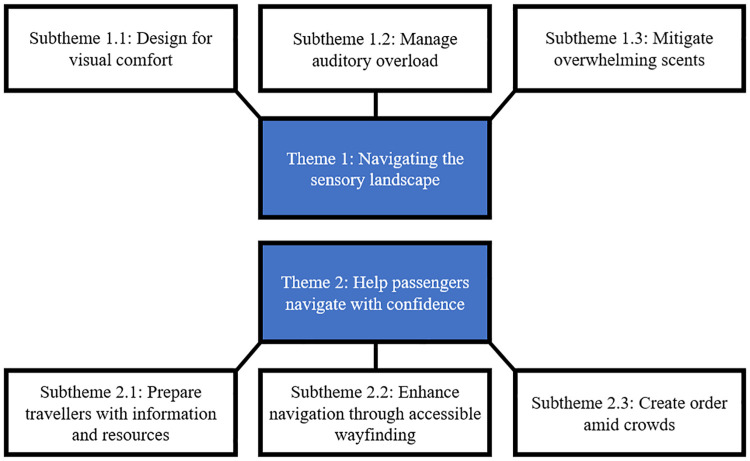
Thematic map based on analysis of airport environmental assessment reports.

Our RTA generated two overarching themes: *Navigating the sensory landscape* and *Help passengers navigate with confidence*. The first theme explores how visual, auditory, and olfactory elements influence the accessibility of airport environments for Autistic travelers, highlighting both barriers and enablers. The second theme emphasizes the importance of clear communication and structured environments, encompassing three subthemes: preparing travelers with information and resources, enhancing navigation through accessible wayfinding, and managing crowds and queues for predictability. Together, these findings underscore the critical role of designing for sensory comfort, accessible communication, and structured systems in fostering inclusive and supportive airport experiences for Autistic people. Figure 1 provides a thematic map illustrating these relationships.

### Theme 1: Navigating the sensory landscape

This theme explores how sensory elements within airport environments—visual, auditory, and olfactory—shape accessibility for Autistic travelers. Findings from the environmental assessment reports reflect the dual role of sensory stimuli, which can act as enablers or barriers depending on the design and management of spaces. In particular, the reports emphasized the value of “quiet rooms and/or sensory room options where people can ‘decompress’ before or after their flight” (Airport 5), highlighting how dedicated spaces for sensory regulation can support Autistic travelers in navigating otherwise demanding environments. This theme underscores the importance of thoughtful sensory design in creating inclusive and supportive airport spaces.

#### Subtheme 1.1: Design for visual comfort

Visual design was consistently highlighted in the reports as an influential factor in sensory accessibility. Positive examples included features such as the wooden ceiling in Airport 1, which “reduce[d] reflections and glare from lighting,” and the carpeting in Airport 6, which “prevent[ed] overhead lights from reflecting off the floor.” These examples demonstrate how intentional design can reduce visual distractions and promote more calming environments. Similarly, natural features, such as the plant wall in Airport 1, were noted for providing “good visual stimulation,” underscoring the benefits of integrating biophilic design elements.

Challenges in managing visual stimuli were widespread throughout reports, with reflective materials such as “glass, tiles, Perspex, and table tops” (Airport 1) frequently identified as problematic. Lighting was another common concern, with green-tinted glass at Airport 2 described as particularly disruptive and “halogen lighting” at Airport 3 noted for its flickering. These elements were associated with sensory discomfort for Autistic travelers. Design recommendations included reducing reflective surfaces, using matte finishes, and replacing existing lighting with “LED lighting that is warm and has diffuser plates” (Airport 1).

The reports also emphasized the importance of visual comfort in sensory-friendly spaces. For instance, a quiet room at Airport 6 was described as having “freshly painted bright white walls,” which made the room feel uncomfortable due to reflections from bright lighting. Recommendations to use “natural or muted colors” (Airport 6) highlight how visual design can significantly influence the effectiveness of sensory-friendly areas.

#### Subtheme 1.2: Manage auditory overload

Alongside visual elements, the auditory environment was commonly described as a significant factor in shaping the accessibility of airport spaces for Autistic travelers, with both positive practices and ongoing challenges identified in the reports. Positive examples highlighted the potential of thoughtful sound management to create calmer, more predictable environments. For instance, “acoustic treatment installed on the ceiling, along with the carpeted floors” (Airport 6) was noted for its ability to reduce noise levels and minimize echoes, fostering a quieter, more manageable atmosphere. Similarly, the inclusion of “airlocks at airport entrances” (Airport 1) was recognized as a practical feature for facilitating smoother transitions between loud and quiet areas, supporting travelers as they adjusted to shifting soundscapes. Calming auditory features, such as a “water feature which gave off a calming sound” (Airport 1), further demonstrated the value of strategic design in promoting a sense of ease and relaxation in otherwise demanding settings.

Despite these promising examples, the reports revealed considerable gaps in auditory management across many airport environments. In particular, the absence of adequate soundproofing was a recurring concern. At Airport 3, “no soundproofing evident” in a “large space with hard reflective surfaces” amplified noise levels even when the space was sparsely populated. Such environments were described as particularly challenging for Autistic travelers, who often experience heightened sensitivity to auditory input. Compounding these difficulties, sudden and unpredictable noises, such as “beeping and loud walkie-talkies used by staff” (Airport 6), were frequently identified as distressing, underscoring the need for greater consideration of the sensory impacts of operational practices.

The reports provided several actionable recommendations to address these issues and create more supportive auditory environments. These included incorporating soundproofing materials to reduce ambient noise, designing quieter spaces, and adopting proactive auditory strategies, such as the “warning sound ahead of announcements” (Airport 2). This simple yet effective measure allows travelers to anticipate potentially loud sensory inputs, reducing the stress associated with unexpected disruptions. Another key recommendation centered on the design of sensory-friendly toilets. The inclusion of facilities in each terminal that “disable automatic hand dryers and provide paper towels” (Airport 6) was highlighted as a practical solution to minimize distress caused by sudden, unavoidable noises in essential spaces. These accommodations extend the principles of sensory relief to facilities that are fundamental to the travel experience, ensuring that they are accessible to all travelers, including those with heightened auditory sensitivities.

#### Subtheme 1.3: Mitigate overwhelming scents

Olfactory stimuli were frequently identified as overwhelming in certain areas of the airports, with duty-free sections noted as unavoidable and particularly challenging due to “strong perfume smells and makeup smells” (Airport 3). These strong scents were recognized as being overwhelming for many Autistic travelers. To address this, some reports suggested creating “a bypass route so that passengers can avoid walking through duty-free” (Airport 2) or designing pathways to minimize exposure to strong, lingering smells.

Toilets were also a common source of olfactory discomfort, with some airports using “very strong air fresheners/chemical smells” (Airport 1), which created an unpleasant sensory experience. Reports consistently recommended the use of “low or no scent air fresheners for toilets” (Airport 6) to balance the need for cleanliness with sensory comfort, ensuring these essential facilities do not contribute to sensory overload.

### Theme 2: Help passengers navigate with confidence

This theme emphasizes the need for accessible communication tools and clear wayfinding systems tailored to the diverse needs of travelers. Findings from the environmental assessments highlighted how well-designed pre-travel resources, signage, and visual aids can reduce anxiety, enhance predictability, and improve the overall travel experience for Autistic people. The recommendations emphasize proactive and inclusive communication strategies to help travelers understand what to expect and what is expected of them.

#### Subtheme 2.1: Prepare travelers with information and resources

Pre-travel resources and accessible communication tools were highlighted as crucial for reducing uncertainty and enhancing preparedness for Autistic travelers. Airports that provided comprehensive and accessible materials were praised for their proactive approach. For instance, Airport 2 was commended for its “dedicated accessibility webpage that is easily found from the landing page” and its “extremely detailed, downloadable accessibility guide outlining all accessibility features and supports available.” This resource also allowed passengers to request a Sunflower lanyard and additional support in advance, ensuring travelers “do not have to rely on verbal communication at the airport,” which was recognized as a significant enabler for reducing stress. Despite some positives, gaps in resources were consistently identified. For example, the website at Airport 6, while seen as a “positive step,” was noted for offering “limited information,” which could cause confusion for some travelers. In addition, Airport 1 was found to lack materials “in Easy English,” limiting the accessibility of essential information for some individuals.

Reports also highlighted the importance of visual aids in providing clarity and reducing anxiety during the travel experience. Effective practices included animations on security screens at Airport 6, which “depict in a simple and clear way what is expected during the screening process.” Similarly, Airport 3 was praised for clear visual instructions that helped travelers “navigate and complete the process independently, without having to ask for help, get things wrong, or become frustrated and overwhelmed.” However, gaps in visual communication were noted across the airports. For instance, at Airport 4, incomplete visual instructions during security screening led to confusion when an Autistic consultant “did not know they had to remove other electronic devices from their bag.” Bathrooms with “two-in-one hybrid taps and hand dryers” (Airport 3) were also flagged as challenging due to “minimal visual instruction,” further illustrating how unclear guidance can increase stress and uncertainty.

#### Subtheme 2.2: Enhance navigation through accessible wayfinding

The importance of clear and accessible wayfinding systems was a recurring focus across the reports, with signage and visual aids identified as critical for reducing anxiety and supporting Autistic travelers. Airports with effective signage were recognized for their role in enabling smoother navigation. For example, newly installed signage at Airport 6 was praised for being “exceptionally clear by using icons alongside text,” and the use of high-contrast colors, such as “clear large yellow and black icons,” at Airport 4 was noted as particularly helpful for Autistic people who “rely on familiar visual cues for navigation and comprehension.” Signage displaying estimated walking times or queue waiting times was also highlighted as beneficial, with one report describing these signs as “incredibly helpful, allowing for better preparation and reducing anxiety about time constraints” (Airport 6).

However, inconsistent or unclear signage was a common challenge in airports. For example, at Airport 1, “there was little to no signage throughout the terminal to help find a toilet,” creating unnecessary stress for travelers trying to locate essential facilities. Similarly, Airport 3 lacked clear signage for accessible public pick-up points, with “no evident signage” guiding travelers to these areas. Reports also flagged issues with signage at critical points, such as security screening, where “no signage or dedicated lane for any accessibility needs traveling through security screening” (Airport 3) left travelers unsure of where to go for support. The recommendations emphasized the need for signage that is consistent, clear, and visually accessible. Airports were encouraged to adopt practices such as using icons alongside text, high-contrast colors, and intuitive layouts to reduce confusion.

#### Subtheme 2.3: Create order amid crowds

Managing crowds and queues is a critical aspect of fostering a more predictable and accessible airport experience for Autistic travelers. The reports highlighted effective strategies, such as the use of retractable barriers and clear signage, which were praised for “creating a clear queuing system and informing people where and how to line up” (Airport 4). These strategies helped establish order, reducing uncertainty and confusion for travelers, particularly those who thrive in structured and well-organized environments.

However, many areas across the airports assessed struggled with overcrowding and disorganized queues, which significantly impacted accessibility. At Airport 3, the absence of a defined queuing system resulted in “people coming from a couple of directions, causing a crowd rather than a queue,” creating a sense of chaos and discomfort. Limited seating availability exacerbated these issues. For instance, at Airport 2, the scarcity of seating at gates led to passengers congregating in other areas, such as food courts, further intensifying crowding and congestion.

The absence of accessible lanes for travelers with hidden disabilities, such as Autistic travelers, was another area for improvement. Reports recommended implementing “a clearly labelled dedicated accessible lane” (Airport 1) at critical points, such as security checkpoints, to provide a less crowded and more supportive option for travelers who may “take extra time to complete the process” (Airport 3). The use of signage indicating the Sunflower symbol was also highlighted as an effective way to ensure travelers could access assistance discreetly, reducing the need for verbal explanations.

To address these challenges, the reports emphasized the importance of revising queuing systems to minimize congestion, increasing seating availability, and enhancing signage to guide travelers effectively. Collaboration with airlines was also suggested, with airports encouraged to “make suggestions to airlines regarding best practice requirements for queuing” (Airport 5) to ensure consistency in accommodating travelers with hidden disabilities.

## Discussion

This study presents a retrospective analysis of Autistic-led environmental assessment reports conducted at six Australian airports between 2017 and 2024. In response to our research question—What are the common accessibility challenges and enablers in airports, as identified through Autistic-led environmental assessments guided by an autism-friendly framework?—we identified two overarching themes: (1) *Navigating the sensory landscape* and (2) *Help passengers navigate with confidence*. These findings provide valuable insights into airport accessibility for Autistic people, highlighting both existing strengths and areas requiring significant improvement. By contributing to the expanding field of autism-friendly design, this research underscores the urgent need for more inclusive, sensory-sensitive approaches in creating accessible environments within high-stress, dynamic settings such as airports. Moreover, our study demonstrates the invaluable role of Autistic perspectives in shaping truly accessible public spaces.

Our findings highlight the profound impact that sensory elements have on the airport experiences of Autistic people, aligning with a broader body of research on sensory experiences in public spaces (e.g. Brake, 2024; Chen et al., 2024; MacLennan et al., 2021; MacLennan et al., 2023; Parmar et al., 2021). The identification of both enabling features (e.g. calming artwork, soothing water features) and disabling elements (e.g. highly reflective surfaces, unpredictable loud noises) underscores the notion that environments are not passive but actively shape the experiences of Autistic people (MacLennan et al., 2023). This spectrum of sensory experiences draws attention to the pervasive role of societal barriers in creating disabling environments for Autistic people (Dwyer, 2022).

Analysis of the reports highlighted several key areas for promoting more positive and reducing negative sensory experiences, particularly regarding lighting, reflection, auditory, and olfactory experiences within airport environments. The preference for indirect, natural lighting over harsh fluorescent options, the need to reduce reflective surfaces, and the recommendation for neutral color palettes (except in signage where contrast aids wayfinding) all point to the ways in which visual stimuli can be improved for Autistic travelers. Similarly, the emphasis on sound-absorbing materials and neutral scents underscores the multisensory nature of airport environments and the potential for sensory overload. These findings not only align with broader recommendations on making environments more sensory-friendly for Autistic people (Black et al., 2022; Doherty et al., 2023; Garner et al., 2024; Mostafa, 2021) but, to the best of our knowledge, mark the first time these observations and recommendations have been systematically applied to the complex context of airport environments.

While aspects of airport environments such as crowd levels and general noise cannot be fully controlled, our research highlights the critical need to provide options for sensory regulation. Among these, designated quiet spaces were identified as a significant finding, offering essential opportunities for Autistic travelers to retreat and recover from sensory overload, making them indispensable components of inclusive airport design (Black et al., 2022; MacLennan et al., 2023). Equally important is facilitating sensory coping mechanisms, such as the use of noise-canceling headphones or glasses, particularly during security procedures. However, the effectiveness of these strategies often depends on the awareness and understanding of security personnel.

Making environments more accessible for Autistic people requires more than just sensory adjustments (Garner et al., 2024; MacLennan et al., 2023; Manning et al., 2023). Our second theme emphasizes the critical role of clear communication and predictable environments in supporting Autistic travelers, particularly as planning and preparation can help reduce uncertainty before encountering challenging sensory environments (MacLennan et al., 2023). This is especially relevant in airport settings, where preparation and predictability are crucial for enabling Autistic people to travel with confidence (Cerdan Chiscano, 2021; Conde et al., 2023; Dempsey et al., 2021; Santos et al., 2024). However, the inconsistency in pre-travel resources across airports reflects a broader issue within tourism accessibility, where the needs of those with hidden disabilities have often been overlooked (Jepson et al., 2024). While the fundamental aspects of air travel remain consistent, every airport is different—featuring unique layouts, terminal-specific airlines, architecture, signage, and procedures—making it impossible to assume that familiarity with one airport equates to understanding them all. Given the complexity and unpredictability inherent in airports—due to fluctuating sensory stimuli, security protocols, and crowd levels—it is essential that airports prioritize the provision of clear, comprehensive, and accessible pre-travel information. While these factors cannot be entirely controlled, equipping travelers with the necessary resources can help mitigate unpredictability and empower Autistic people to navigate these dynamic spaces more confidently.

Intuitive wayfinding systems and visual supports were also highlighted as essential elements of autism-friendly design. While intuitive wayfinding is essential in any built environment (Black et al., 2022), it becomes particularly significant for individuals with hidden disabilities, such as autism, in complex and often overwhelming settings like airports (Santos et al., 2024). Visual supports—such as clear signage, color-coded pathways, and pictorial instructions—are not only valuable for improving spatial orientation but also serve as alternative modes of communication, which are crucial for enhancing accessibility for Autistic people (Garner et al., 2024). Importantly, these visual supports must accurately reflect the processes they are meant to guide, as inconsistencies can cause uncertainty and confusion rather than clarity. In our view, ensuring that wayfinding systems are both accessible and intuitive across all areas of an airport can help make these spaces more inclusive and navigable—not only for Autistic travelers but also for the wider public—creating environments that are supportive and accessible for everyone.

While environmental modifications are essential, their success can heavily depend on having well-trained airport personnel who understand neurodiversity and can appropriately support Autistic travelers, as unsupportive staff makes it difficult to access support (MacLennan et al., 2023; Santos et al., 2024). Trainers should include lived experience and cover fundamental aspects of autism awareness, sensory differences, communication preferences, and crisis management strategies. Particularly important is ensuring that security personnel understand how to conduct necessary procedures while respecting sensory needs and allowing for adaptive equipment like noise-canceling headphones when possible. To further enhance the inclusivity of airport environments, multi-disability environmental walkthroughs could be conducted, involving representatives from diverse disability groups. These walkthroughs can provide valuable insights into the overlapping and possibly conflicting needs of travelers with varying disabilities, helping to guide more holistic staff training and environmental modifications. Such initiatives and training need to be ongoing and systematically integrated into airport operations rather than treated as a one-time initiative, reflecting the dynamic nature of airport environments and the diverse needs of all travelers.

### Limitations

While this study provides important insights into the accessibility of airports for Autistic travelers, several limitations must be considered. The shared organizational affiliation between the Autism Friendly team and ARCAP, while mitigated by independent analysis, may introduce potential bias, and this relationship should be borne in mind when interpreting the findings. In addition, the study primarily relied on a pool of four Autistic consultants to conduct the assessments, which, while ensuring consistency, may have contributed to recurring themes that reflect their shared perspectives. However, the use of the structured Aspect Autism Friendly Framework minimized the influence of individual interpretations by providing a replicable and systematic methodology. Furthermore, only the Head of Autism Friendly and one experienced consultant contributed to the write-up, as member-checking with all consultants was not feasible due to part-time roles and billable workloads. A further limitation is that the autism-friendly framework has not been peer-reviewed and it has evolved over the years, incorporating feedback and the latest research, which made the more recent airport assessments more comprehensive compared to earlier ones. This progression may have influenced the richness of data across airports, potentially favoring more detailed findings from recent assessments. Although the study identifies common themes across six airports, each airport’s unique design, layout, and operations mean that site-specific assessments and tailored staff training are still necessary. Furthermore, the consultants’ insights, while invaluable, do not encompass the full diversity of Autistic experiences, particularly those of individuals with varying support needs and travel histories.

### Future research

Future research should focus on hearing from a broad range of Autistic people, including children and their families, about their experiences traveling through airports that have implemented autism-friendly initiatives. This would provide valuable insights into how various environmental features impact the travel experience for Autistic people. In addition, as noted by Jepson et al. (2024), while the tourism industry and airports must make structural adjustments to improve accessibility, an effective approach to airport accessibility requires shared responsibility. This involves both implementing changes within the tourism system and empowering individual strategies to enhance overall accessibility. Future research should therefore consider a coordinated approach that spans the entire air travel journey—including the roles of travel agents, airports, and airlines—to create a seamless, autism-friendly experience from start to finish.

Moreover, future studies should expand beyond Australia and include airports globally to capture a diverse range of perspectives and practices. Research should explore whether autism-friendly airport initiatives can successfully support Autistic people who have previously avoided air travel due to the associated challenges. Understanding if autism-friendly initiatives enable those who have been hesitant to travel to feel comfortable enough to engage with air travel would be a key indicator of the effectiveness of these efforts. Longitudinal research examining the impact of implemented changes on Autistic travelers’ experiences would also be valuable, as would studies exploring the perspectives of diverse Autistic people across different age groups and support needs.

## Conclusion

This study marks a significant step toward improving the accessibility of Australian airports for Autistic travelers. Through a retrospective analysis of Autistic-led environmental assessments, we have highlighted the critical importance of sensory adaptations, accessible communication and visuals, and effective crowd management in creating more autism-friendly airport environments. These findings contribute to the growing recognition that accessible design is not a matter of preference but a necessity for ensuring equitable participation in public spaces.

As the tourism and travel industries continue to evolve, it is essential that airports adopt a proactive approach to accessibility, prioritizing the voices and experiences of Autistic people. By embedding inclusive design principles into the core of airport planning and operations, airports can become more than just transit hubs—they can be welcoming, supportive spaces for all travelers. Ultimately, this research underscores the transformative potential of participatory, Autistic-led initiatives in driving positive change and fostering environments that accommodate the diverse needs of the traveling public.

## Supplemental Material

sj-docx-1-aut-10.1177_13623613251337200 – Supplemental material for Autistic-led insights on airport accessibility: A retrospective analysis of environmental assessments Supplemental material, sj-docx-1-aut-10.1177_13623613251337200 for Autistic-led insights on airport accessibility: A retrospective analysis of environmental assessments by Chris Edwards, Abigail MA Love, Ru Ying Cai, Tom Tutton, Emma Beardsley and Vicki Gibbs in Autism

## Footnotes

1. We have intentionally chosen to capitalize the term ‘Autistic’ to acknowledge its role as a significant and valued identity. This decision reflects the importance of recognizing Autistic people as part of a cohesive and empowered community.
