# Supplementary material for: Autistic-led insights on airport accessibility: A retrospective analysis of environmental assessments
Source: Autism. 2025 May 9;29(8):2151–62. doi: 10.1177/13623613251337200 (PMC12255834; doi:10.1177/13623613251337200)
Supplement: sj-docx-1-aut-10.1177_13623613251337200 – Supplemental material for Autistic-led insights on airport accessibility: A retrospective analysis of environmental assessments [file sj-docx-1-aut-10.1177_13623613251337200.docx]

**Supplemental Material – Aspect Autism Friendly Framework**

This document provides additional context about the Aspect Autism Friendly Framework and the processes used during the environmental assessments, offering transparency regarding how the data analyzed in this study were produced. The Aspect Autism Friendly Framework is included here to clarify the structure that informed data collection, though it was not used to guide the data analysis directly.

**Framework Development**

The Aspect Autism Friendly Framework was developed through a co-design process involving Autistic consultants, researchers, and practitioners. This iterative process incorporated lived experience, evidence-based practices, and stakeholder feedback to ensure the framework was practical, accessible, and reflective of Autistic perspectives. It has continued to evolve over time, remaining adaptable and informed by current research and best practice. The framework draws on peer-reviewed literature (e.g., Autistic SPACE, Doherty et al., 2023) and has been applied across a range of real-world settings, including airports, trains, musicals, museums, sports venues, and retail environments.

**Eight Elements of the Aspect Autism Friendly Framework**

The framework is organized around eight key elements that emphasize creating environments that are predictable, structured, and supportive of sensory and communication differences:

1. **Culture of inclusion**: Explicitly and visually make people feel welcome, understood, valued, and that they belong without needing to change who they are to fit in.
2. **Preparation and predictability**: Provide information about what people can expect and what they are expected to do, such as unwritten rules and expectations (hidden curriculum).
3. **Structure and visual organization**: Help people visually and intuitively understand and navigate their surroundings and processes.
4. **Sensory adaptations**: Reduce challenging sensory experiences, provide positive ones, forewarn of potential challenges and support coping (e.g. quiet rooms).
5. **Communication supports**: Adapt communication to the method that works best for the person, provide visual supports and resources, and allow time for processing.
6. **Proactive problem solving**: Anticipate potential stressors or problems that might arise and find ways to minimize risk.
7. **Staff training**: Ensure all staff receive strengths-based, positive training co-presented with an Autistic person. This training should describe how Autistic people experience the world and offer practical solutions for support.
8. **Review and improve**: Develop feedback mechanisms and meaningfully act on feedback to ensure continuous improvement.

**Training and Internal Consistency**

All consultants receive training on the elements of the Aspect Autism Friendly Framework and on-the-job training conducting assessments. This consists of observing experienced consultants, then completing assessments alongside them, before conducting their own assessments with feedback and supervision. This approach helps build capacity and ensure fidelity to the framework across team members. Reports are subject to internal peer review by other members of the team before being finalized and delivered to clients. This collaborative review process helps ensure consistency in tone, scope, and the application of the framework across different environments.

**Airport Assessments Using the Aspect Autism Friendly Framework**

Assessments were conducted as in situ walkthroughs of the airport environment by two consultants (at least one Autistic), simulating the journey of a passenger from pre-travel (e.g., website navigation) through to boarding or arrival. The framework was actively used by consultants throughout the airport assessments, guiding what was observed and how feedback was recorded. Observations were recorded contemporaneously using digital note-taking, audio recording and photographs, guided by the framework’s eight elements, which were kept front of mind during the walkthrough. No formal observation forms or rating scales were used, however the simplicity and flexibility of the framework enabled consultants to reflect on each of the eight elements during the walkthrough. Observations were later structured in the reports using either the eight framework elements or the stages of the passenger journey. This flexible but consistent approach allowed consultants to tailor their observations to each airport while ensuring that all key accessibility domains were addressed.

**Aspect Autism Friendly Framework in Research and Practice**

Although this study represents the first formal peer-reviewed publication describing the Aspect Autism Friendly Framework in detail, its principles have been applied in multiple settings. A peer-reviewed tourism study (Edwards et al, 2024) explored the experiences of Autistic adults and parents of Autistic children at two Australian airports that had implemented recommendations based on two of the six environmental assessment reports analysed in this study. These initiatives included the introduction of sensory maps, visual journey planners, social stories, quiet rooms, improved signage, and participation in the Hidden Disability Sunflower program. The study found that these initiatives had a meaningful impact: participants described the airports as notably more accessible and inclusive than others they had encountered, and many reported feeling recognized, supported, and more at ease while travelling. At the same time, the study highlighted areas for ongoing improvement, including the need for greater visibility of accessibility supports, proactive assistance from staff, and standardization of training. The research offers some evidence for the practical value of the Aspect Autism Friendly Framework and reinforces the importance of participatory, lived-experience-informed approaches to inclusive design.

The Aspect Autism Friendly Framework has also been applied in broader public-facing initiatives. A notable example is the 2024/2025 relaxed performance of Disney’s Beauty and the Beast, produced in partnership with Aspect. Two of Aspect’s Autistic consultants assessed the production and made targeted recommendations based on the framework. These included the development of a step-by-step visual story, the use of inclusive language during ticketing and communications, and logistical modifications such as turning off announcements, providing multiple quiet spaces, and ensuring the presence of additional trained staff and volunteers. Adjustments to the production itself included dimmed house lights, reduced auditory intensity, and visual cues to signal potentially intense moments—strategies aligned with the Aspect Autism Friendly Framework’s elements of sensory adaptations, preparation and predictability, and proactive problem solving. A short pre-show welcome was also developed to familiarize the audience with key characters and sensory features, demonstrating the framework’s flexibility in supporting inclusive experiences across highly sensory and dynamic environments.

**Further Information**

A public brochure outlining Aspect’s Autism Friendly work, the framework’s application, and client partnerships is available, alongside further information on the organization’s website (<https://www.aspect.org.au/our-services/autism-friendly>).
